# A Receptor’s Tale: An Eon in the Life of a Trypanosome Receptor

**DOI:** 10.1371/journal.ppat.1006055

**Published:** 2017-01-26

**Authors:** Matthew K. Higgins, Harriet Lane-Serff, Paula MacGregor, Mark Carrington

**Affiliations:** 1 Department of Biochemistry, University of Oxford, Oxford, United Kingdom; 2 Department of Biochemistry, Cambridge, United Kingdom; Boston College, UNITED STATES

## Abstract

African trypanosomes have complex life cycles comprising at least ten developmental forms, variously adapted to different niches in their tsetse fly vector and their mammalian hosts. Unlike many other protozoan pathogens, they are always extracellular and have evolved intricate surface coats that allow them to obtain nutrients while also protecting them from the immune defenses of either insects or mammals. The acquisition of macromolecular nutrients requires receptors that function within the context of these surface coats. The best understood of these is the haptoglobin–hemoglobin receptor (HpHbR) of *Trypanosoma brucei*, which is used by the mammalian bloodstream form of the parasite, allowing heme acquisition. However, in some primates it also provides an uptake route for trypanolytic factor-1, a mediator of innate immunity against trypanosome infection. Recent studies have shown that during the evolution of African trypanosome species the receptor has diversified in function from a hemoglobin receptor predominantly expressed in the tsetse fly to a haptoglobin–hemoglobin receptor predominantly expressed in the mammalian bloodstream. Structural and functional studies of homologous receptors from different trypanosome species have allowed us to propose an evolutionary history for how one receptor has adapted to different roles in different trypanosome species. They also highlight the challenges that a receptor faces in operating on the complex trypanosome surface and show how these challenges can be met.

## Living in the Hinterland

Receptor proteins of a persistent extracellular pathogen, such as the African trypanosome, inhabit the hinterlands. Outside the safety and protection of the cell body, they experience an environment full of opportunity but where opportunity is matched with danger. The blood and tissue fluids of a mammalian host, or the tissue and blood meals of the biting insects that they inhabit, are nutrient-rich, and receptors are bathed with molecules of great value to the parasite. However, these parasites are also vulnerable and potentially under attack by the immune system of the host and, if detected, risk destruction. The receptors are, therefore, under pressure at the molecular and cellular level to retain the capacity to bind their ligands while avoiding the consequences of recognition by the host.

**Fig 1:**
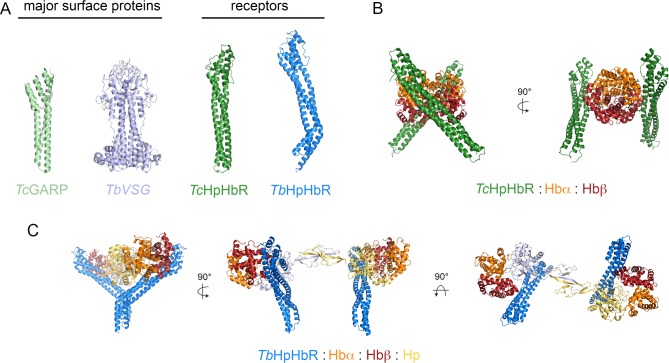
The structures of trypanosome surface proteins. **A.** The structures of the major surface proteins of the *T*. *congolense* epimastigote, the glutamic acid rich protein (GARP) and of the *T*. *brucei* bloodstream form, the variant surface glycoprotein (VSG), and the structures of the *T*. *brucei and T*. *congolense* haptoglobin–hemoglobin receptors (TbHpHbR and TcHpHbR). Both TbHpHbR and TbVSG are elongated by additional C-terminal domains, which are not represented [10]. **B.** The structure of a complex of two TcHpHbR bound to a single hemoglobin tetramer. The receptors are coupled to the cell membrane by a GPI anchor and will tilt in order to simultaneously bind to a single hemoglobin. **C.** The structure of a complex of two TbHpHbR bound to a haptoglobin–hemoglobin tetramer (silver and gold), showing how the kink in the receptor allows two membrane-linked TbHpHbR to simultaneously bind to a single HpHb.

It is this context in which the haptoglobin–hemoglobin receptors (HpHbR) of the African trypanosomes operate. HpHbR first gained fame as the primary uptake route for trypanolytic factor-1 (TLF-1) [1], one of the innate immunity factors in serum responsible for rendering the majority of African trypanosomes unable to infect humans and some other primates [2–8]. This lytic factor contains a pore-forming component, ApoLI, which kills trypanosomes [9] as well as the haptoglobin-related protein, Hpr, which provides an uptake route for TLF1 through its interaction with HpHbR [1]. HpHbR is the best understood trypanosome receptor, and a recent series of studies allows us to propose how it has evolved during trypanosome speciation—changing structure, ligand-specificity, and expression pattern to adapt to different roles [10–14] (Fig 1).

## An Ancient Receptor with a Changing Role

HpHbR was first identified in *Trypanosoma brucei* and its two human infective subspecies, *T*. *b*. *rhodesiense* and *T*. *b*. *gambiense* [1]. In these trypanosomes, the receptor is expressed in the proliferative slender bloodstream form of the parasite, in which it mediates the acquisition of heme [1]. Under normal circumstances, human blood contains little free hemoglobin (Hb), as Hb released from damaged erythrocytes is detoxified through interaction with haptoglobin (Hp) to form haptoglobin–hemoglobin (HpHb) complexes [15, 16]. These are then cleared from the blood through endocytosis by macrophages via the CD163 receptor [16]. *T*. *brucei* HpHbR (TbHpHbR) binds specifically to HpHb, allowing the cell to scavenge heme [1]. However, TbHpHbR has no appreciable affinity for free Hb [1], which is generally present at only negligible levels in the blood.

As all African trypanosomes share the capacity of *T*. *b*. *brucei* to live in the blood of their various mammalian hosts, it was assumed that HpHbR would have a similar role across species. However, a recent discovery revealed that HpHbR in the common cattle pathogen *T*. *congolense* has very different properties [12, 14]. Firstly, the *T*. *congolense* receptor has different ligand specificity, binding to free Hb with an affinity some 1,000-fold stronger than its affinity for HpHb [12, 14]. Secondly, it has a changed expression profile. Analysis of *T*. *congolense* bloodstream form cells have shown no detectible levels of HpHbR RNA [14] or protein [12, 14], and no uptake of Hb was observed into these cells, indicating that the receptor is present at a very low copy number, if at all [14]. Experiments with transgenic mice do, however, imply the presence of some HpHbR in the blood stage of *T*. *congolense*. The growth of *T*. *congolense* in mice transgenic for the trypanolytic pore-forming protein, ApoLI, is significantly reduced by the additional expression of the haptoglobin-related protein, Hpr [17]. As HpHbR is the primary route for the high efficiency uptake of Hpr-containing complexes into trypanosomes [1], this suggests the presence of sufficient HpHbR in *T*. *congolense* bloodstream forms to allow uptake of enough ApoLI to kill the cells while not being detectable in other assays. In contrast to these low expression levels, the receptor is expressed to extremely high levels in the *T*. *congolense* epimastigote, a late developmental form found in the mouthparts of the tsetse fly, allowing uptake of Hb [12, 14, 18]. *T*. *congolense* HpHbR (TcHpHbR) is therefore primarily a hemoglobin receptor that is used as the trypanosome inhabits its insect vector.

So, what came first: an HpHb receptor expressed primarily in the bloodstream form or an Hb receptor expressed primarily in the insect? A clue came from studying the receptor from *T*. *vivax*, which diverged from the common ancestor earlier in evolutionary history than the divergence of *T*. *congolense* and *T*. *brucei* [19, 20]. The discovery that the *T*. *vivax* HpHbR is also an Hb receptor [12] that is expressed primarily in epimastigotes [21] provides strong evidence for the view that this is the ancestral form of HpHbR and that later evolutionary changes resulted in a bloodstream form HpHb receptor in *T*. *brucei* [12].

Our knowledge of the structure and function of HpHbR from these parasite species allows us to propose some of the evolutionary adaptations that have taken place as the receptor has changed its location and role. This also illustrates some of the pressures experienced by a receptor of the trypanosome hinterlands and identifies some general principles that are likely to affect how such a receptor operates.

## The Ancestral Receptor—Lessons from *T. Congolense*

**Fig 2:**
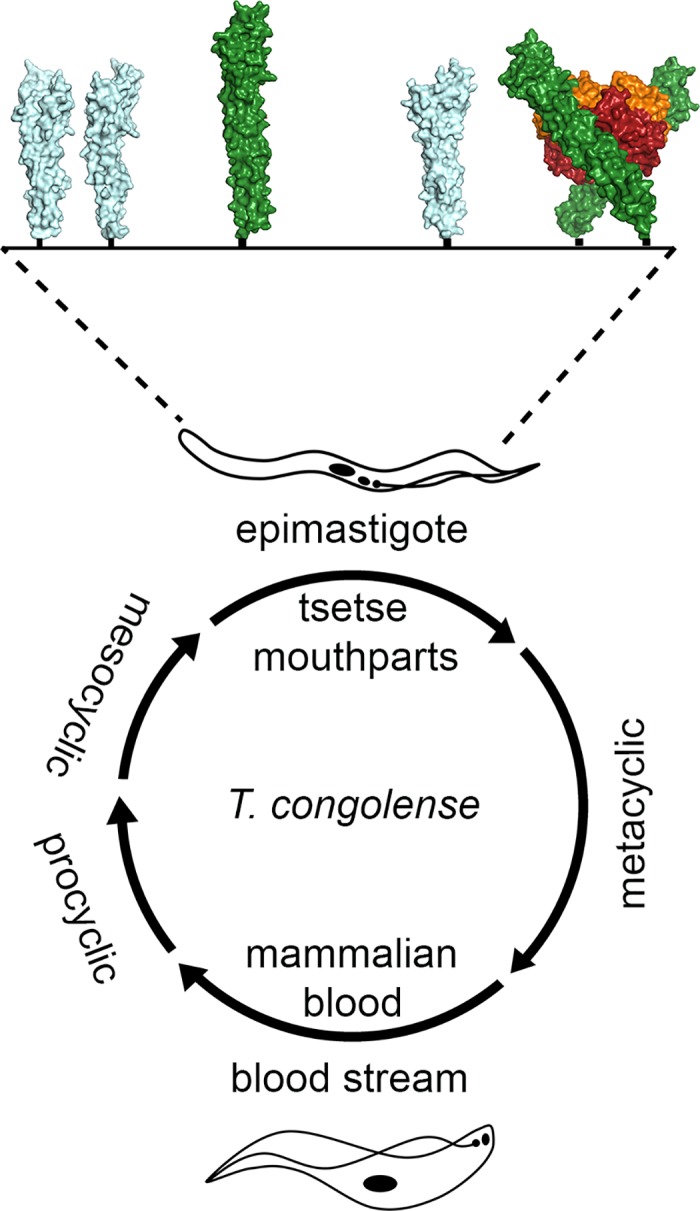
The *T*. *congolense* HpHbR is an epimastigote expressed Hb receptor. In the life cycle of *T*. *congolense*, HpHbR is expressed predominantly in the epimastigotes that inhabit the mouthparts of the tsetse fly, where it binds to Hb present in the blood meal of the fly. Here the receptor functions in the context of the major epimastigote surface protein, GARP. In the structure figure TcHpHbR is green, GARP is light blue, and Hb is orange and red.

The epimastigotes of *T*. *congolense* inhabit the mouthparts of tsetse flies, adhering tightly to the chitin-rich surfaces of the cibarium and proboscis [22–24] (Fig 2). In this location, they are likely to be exposed to any Hb that has been released from lysed red cells of an incoming blood meal as it passes towards the tsetse midgut or as it passes back from the midgut to the mouthparts, as the tsetse fly regurgitates gut contents during feeding. Both will allow the acquisition of heme through binding and internalization of this Hb. Because these opportunities are fleeting, the parasite has ensured that it is poised for feeding time with the expression pattern and location of the receptor both optimized for this role. Firstly, the receptor is highly expressed, with around half a million copies per cell [12], which is three orders of magnitude greater than that found on bloodstream form *T*. *brucei* [1, 25]. Secondly, HpHbR is evenly distributed over the entire epimastigote surface [12]. This will increase its opportunity to interact with, and capture, hemoglobin, followed by stochastic diffusion to the flagellar pocket for subsequent uptake. The mobilization of the receptor in this way allows the parasite to take advantage of the transient nature of its exposure to ligand, perhaps allowing it to acquire and store sufficient heme to last until the next blood meal.

The structure of the receptor is also well suited to allow it to operate in the context of other surface molecules. While the protein density of the epimastigote cell surface has not been quantitatively characterized, electron microscopy images suggest that these cells lack the dense protein coat observed in bloodstream form trypanosomes [26]. TcHpHbR is an elongated three-helical bundle [10], ~25% longer than the predominant epimastigote surface molecule, glutamic-acid rich protein (GARP) [10, 27]. The Hb binding site lies on the side of the receptor at its membrane distal end [12] and is therefore expected be accessible to ligand despite the other molecules of the epimastigote surface coat. In addition, the mode of attachment of the receptor to the membrane surface, through a flexible GPI-anchor at the C-terminus, is expected to allow it to move laterally across the membrane surface, as seen for the variant surface glycoproteins (VSGs) [28], and also to adopt a significant tilt angle. This will allow two receptors to simultaneously bind to a single hemoglobin tetramer, further increasing the avidity for the ligand and uptake into the parasite [12] (Fig 1). The lateral mobility allowed by the GPI-anchor will also give the receptor-Hb complex freedom to diffuse across the cell until it reaches the flagellar pocket, an active site of endocytosis, from which it will be internalized [29].

## The Switch to the Blood Stage—A Complex Story of Molecular Adaptation

**Fig 3:**
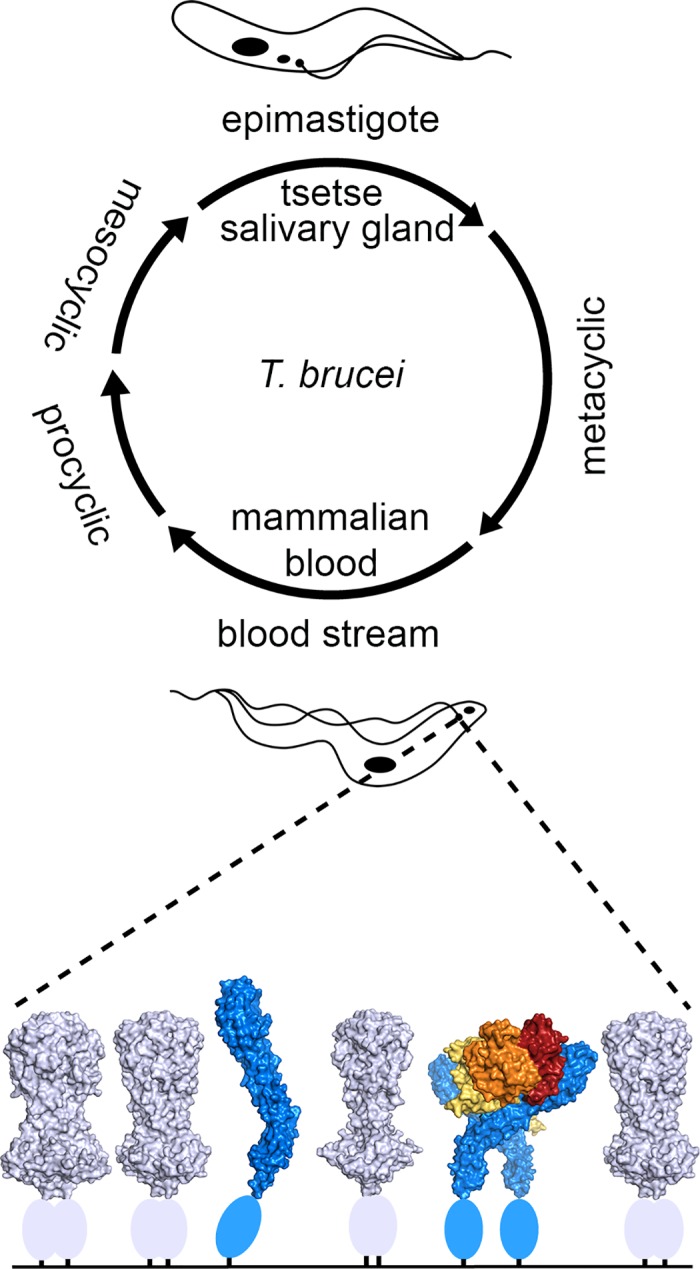
The *T. brucei* HpHbR is a bloodstream form HpHb receptor. In the life cycle of *T*. *brucei*, the epimastigotes inhabit the salivary glands of the tsetse fly and do not express HpHbR. Instead, TbHpHbR is predominantly expressed in the bloodstream form, in which it acts as an HpHb receptor that exists within the densely packed VSG layer. In the structure figure HpHbR is blue, VSG is blue-white, and HpHb is yellow, orange, and red. The ovals represent the C-terminal domains of the VSG and HpHbR. These lie between the N-terminal domains and the membrane, but their relative locations are uncertain.

In *T*. *brucei*, a major transition appears to have taken place in the structure and function of HpHbR, with the receptor now employed primarily as a bloodstream-expressed HpHb receptor [1] (Fig 3). The reasons for this are only partly understood, but the transition may have begun when the epimastigotes changed their preferred location within the tsetse fly. Instead of being located in the mouthparts of the fly, the epimastigotes of *T*. *brucei* are found within the salivary glands [22–24]. It is possible that moving to this new location provided them with a niche not occupied by other trypanosomes. Alternatively, it may have increased their transmission to their mammalian host. Either way, this change rendered the receptor redundant, as epimastigotes in the salivary glands are no longer exposed to Hb from the blood meal. Instead, the receptor became primarily expressed in the bloodstream form, in which it can support heme acquisition from the blood [1]. This is clearly advantageous, as demonstrated by the reduced virulence that results from deletion of the receptor in *T*. *brucei* [1]. However, the bloodstream form trypanosome surface provided a new and challenging context for receptor function, which necessitated changes in both its structure and cellular distribution.

In a number of respects, ancestral HpHbR was well suited to its new role as an HpHb uptake receptor. *T*. *congolense* HpHbR binds HpHb complexes [10], albeit with ~1,000-fold lower affinity than Hb, perhaps allowing it to also scavenge HpHb from the blood meal [12]. Indeed, the mechanism of binding Hb to the receptor, in which the receptor interacts with two adjacent Hb subunits [12], will give it the capacity to interact with HpHb even if the Hp chain does not directly interact with the receptor. The receptor could therefore immediately be mobilized for its new function, as its affinity for HpHb is sufficient to operate at the physiological concentrations of HpHb in sera. However, the bloodstream trypanosome surface is very different from that of the epimastigote. Unlike the tsetse fly, mammalian blood is the site of an immunoglobulin-based acquired immune system, and bloodstream form trypanosomes have evolved a cell surface coat of VSG molecules at close to the maximum possible packing density as a counter measure [30–32]. When combined with a system of antigenic variation, this allows a successful population survival mechanism to allow the cell to avoid immune clearance [33]. This provides an unusual context in which the receptor must function. In addition, the presence of immunoglobulins has a second consequence—a high copy number receptor distributed across the cell surface would be vulnerable to detection, putting the trypanosome at risk of destruction. A number of adaptations have therefore occurred to better equip *T*. *brucei* HpHbR to function in the context of the bloodstream form surface.

Firstly, the structure of the receptor has altered to allow it to bind to its ligand more efficiently, increasing the affinity for HpHb by around 10-fold relative to that of the *T*. *congolense* receptor. A comparison of the structures of HpHbR from *T*. *congolense* and *T*. *brucei* suggests that this increase in affinity has resulted from subtle changes to the path of the helical bundle, causing it to alter in curvature to allow closer interaction with the haptoglobin chain of HpHb [11, 13]. This gain in affinity for HpHb has been coincident with a total loss of affinity for Hb [1, 12] after the removal of any selection pressure to retain Hb binding in the absence of free Hb to scavenge from the blood.

Secondly, with the HpHb binding site lying on the side of the receptor, the dense VSG coat would make it inaccessible to HpHb if the receptor retained the structure seen in *T*. *congolense*. The *T*. *brucei* receptor has gained a C-terminal domain, making it longer than the VSG molecules and raising the ligand-binding site further away from the plasma membrane. It has also evolved a ~50° kink in its three-helical bundle [11, 13]. This kink will push apart VSG molecules on either side of the receptor and orient the HpHb binding surface so that it faces the external milieu, ready to bind HpHb. The kink also allows two receptors to bind to a single HpHb dimer without significant tilting of the receptors at the membrane attachment site—something that will be impossible in the densely packed VSG coat. This bivalent binding is important for efficient ligand uptake as the affinity of the receptor for HpHb monomers is only ~1 μM, and only dimeric ligand uptake is efficient in live cells [11]. The receptor has therefore adopted several structural changes to allow it to function in its new location.

Finally, the shift to expression in the bloodstream form has caused changes in both the expression levels and surface distribution of the receptor. While the opportunity for an epimastigote to acquire Hb from a blood meal is fleeting, the bloodstream form inhabits an environment with a more constant supply of HpHb and does not require a high receptor surface density for occasional gorging in the brief times of plenty that occur during a tsetse blood meal. In the blood, the presence of antibodies provides a selection pressure to reduce receptor expression in order to avoid detection and immune clearance. The same pressure has also affected the position of the receptor on the cell surface. While the epimastigote receptor is evenly distributed across the cell surface [12], in the bloodstream form it is concentrated in the flagellar pocket [1], a region of the cell surface that is a site of active endocytosis with reduced accessibility to immune effector cells [29]. This reduced receptor density, coupled with the majority of the receptors being localized to the flagellar pocket, will protect the cell from detection within the mammalian host while allowing it to continue to acquire sufficient heme.

## A Receptor Evolving Away from a New Threat

The evolution of the receptor did not finish with its adaption in *T*. *brucei* to a bloodstream form protein with an affinity for HpHb, as it experienced a new selection pressure from a subset of its mammalian hosts. The majority of species of trypanosomes are not able to infect humans or other primates due to the presence of the trypanolytic factors [6]. These complex particles contain a primate-restricted lytic factor, apolipoprotein LI (ApoLI), which, when taken up into the lysosome of the trypanosome, causes cell death [9, 34]. In addition to ApoLI, the trypanolytic factors contain a variant of haptoglobin, the haptoglobin-related protein (Hpr), bound to Hb [35]. HprHb is recognized by *T*. *brucei* HpHbR, providing a route for the uptake of trypanolytic factors into the cell [1, 11]. This places the trypanosomes in a dangerous situation, with the same receptor providing an important nutrient but also leaving them susceptible to attack by factors of the host innate immune system.

Two subspecies of *T*. *brucei* have evolved the capacity to survive in humans and other primates. *T*. *b*. *rhodesiense* has a single molecule, SRA, which binds to and inactivates ApoLI [9, 36]. In contrast, *T*. *b*. *gambiense* has a more complex mechanism for resistance involving multiple factors, several of which relate to changes in HpHbR [10, 37–39]. Type 1 *T*. *b*. *gambiense* HpHbR has evolved a single point mutation, L210S [10, 40, 41]. This change to a residue that lies adjacent to the HpHb binding site is predicted to disrupt the structure of the receptor and affect HpHb binding [10, 11]. Indeed, this polymorphism results in reduced affinity for both HpHb and TLF1 [10]. This has a greater effect on TLF1 binding than HpHb binding [10]. This may be because the HpHb is dimeric, due to a disulphide-linked dimer, while the Hpr component of TLF1 lacks this disulphide bond, perhaps reducing the percentage of dimeric molecules in the TLF1 particle [42]. Alternatively, it is possible that the insertion of Hpr into TLF1 particles reduces its accessibility to receptor binding.

An additional protective change in *T*. *b*. *gambiense* is the expression of HpHbR at lower levels than the corresponding receptor in *T*. *brucei* [39], and the combination of lower expression levels and reduced ligand affinity decreases the uptake of TLF1 into cells [39]. Coupled with additional factors such as altered cysteine protease activity and the action of TgsGP, which increase the resistance of cells to the smaller amount of internalized trypanolytic factor [37, 38], this allows the parasite to survive in human serum, albeit with potential consequences for its rate of growth [39]. Therefore, HpHbR continues to evolve with its mammalian hosts as it meets the balance between acquiring sufficient nutrients and surviving in an environment in which it is targeted by mechanisms of innate immunity.

## Is HpHbR a Paradigm for Trypanosome Receptors?

HpHbR is the best studied of the trypanosome receptors, and our knowledge of how it operates in both *T*. *congolense* epimastigotes and *T*. *brucei* bloodstream form cells allows us to develop principles that are likely to be followed by other such molecules. It highlights the versatility of the three-helical bundle architecture and shows that receptors can operate in pairs to make uptake more efficient (Fig 1). In addition, adaptations that have taken place to allow the receptor to operate effectively in different trypanosome developmental forms give insight into the surface architecture of these cells and the constraints that they impose on receptor function.

The three-helical bundle architecture is found in many of the cell surface proteins of African trypanosomes (Fig 1). The simplest examples are GARP [27] and HpHbR from *T*. *congolense* [10]. In each case, an elongated three-helical bundle is capped with a small membrane distal head structure, with the major difference being in their length. GARP, which does not have any known binding partner, is around 80% of the length of HpHbR [10], with the greater length of the receptor perhaps allowing it greater access to its ligand. An adaptation to this is seen in *T*. *brucei* HpHbR, in which a change in the placement of the hydrophobic residues that line the core of the helical bundle leads to a ~50° kink that aids its function in the crowded bloodstream trypanosome surface [11, 13]. Alternative adaptations are seen in the VSGs, with the third helix and the membrane distal head becoming decorated with extensions and loops [43–45]. This is a response to the exposure of the VSGs to the mammalian acquired immune system, which provides a route to VSG diversification, creating a wide range of VSGs to allow a system of antigenic variation and immune evasion [46]. Unlike GARP and HpHbR, VSG has also become dimeric, with the first and second helices forming the dimerization interface [43]. Finally, at least three proteins with different functions have arisen from diversification of VSG proteins: the heterodimeric transferrin receptor [47, 48] and two proteins associated with counteracting human innate immunity factors, SRA [36, 49] and TgsGP [37, 38, 50]. These are all currently of unknown structure. This wide range of molecules with different structures and functions shows the deep versatility of the three-helical bundle architecture to adaptation and embellishment [46]. It will be no surprise to discover other trypanosome surface proteins, built from a three-helical bundle, with novel functions.

Studies of HpHbR also show that trypanosome receptors can work in pairs. The affinity of a single *T*. *brucei* HpHbR for HpHb is moderate at ~1μM with a rapid off rate. Indeed, the narrow, elongated structure of the receptor presents a relatively small surface for ligand binding, making it more challenging to develop a high affinity interaction [11, 13]. In the case of HpHbR, this is overcome by the use of bivalent ligand binding. The attachment of receptors to the cell surface through GPI-anchors allows flexibility in lateral movement and also allows receptors to tilt relative to the membrane plane. Two receptors will therefore come together in an appropriate orientation to bind a single ligand. In *T*. *brucei* bloodstream forms, in which dense packing of the VSG coat makes this less likely, the receptor has developed a kink to allow bivalent ligand binding without the receptors adopting a high tilt angle. These approaches dramatically increase the apparent affinity for the ligand and allow dimeric HpHb uptake at reduced concentrations in live trypanosomes [11]. Receptor dimerization to give improved ligand uptake may also be applied in other cases but will only be successful for ligands that have two equivalent binding sites, such as Hb tetramers or HpHb. A natural extension of this, to non-symmetrical ligands, is the assembly of heterodimeric receptors. An example of this may be the transferrin receptor of *T*. *brucei*, which is a heterodimer of ESAG6 and ESAG7 with a structure predicted to resemble a VSG dimer [47]. Further studies will show whether dimerization, either mediated by the ligand or before ligand binding, is widespread in other trypanosome surface proteins.

Structural studies of TbHpHbR have also challenged dogmas about the organization of the bloodstream trypanosome surface. Models assumed that the VSG layer would extend above receptor proteins, covering and protecting them from detection by immunoglobulins. The finding that *T*. *brucei* HpHbR is longer than a VSG showed instead that the receptor protrudes above the surface of the VSG layer [10]. Indeed, ligands for HpHbR are large macromolecular complexes such as HpHb (~150 kDa) [15], TLF1 (~500 kDa), and TLF2 (~1000 kDa) [3, 4], and, on reflection, it is not surprising that the receptor must be readily accessible, as such large ligands would not be able to penetrate the densely packed VSG layer. The use of elongated receptors is not the only strategy used to ensure that they are accessible to large macromolecular ligands. The *T*. *brucei* transferrin receptor is predicted to be similar in size and structure to the VSGs, and it is proposed that extensive glycosylation of the receptor on the surfaces facing the VSG molecules will hold them apart, spreading out the VSG layer and thereby increasing accessibility of the receptor surface to serum ligands [51]. The kink in the *T*. *brucei* HpHbR provides another mechanism by which VSG molecules can be forced apart to leave ligand-binding sites accessible [11, 13]. It will be no surprise to discover that other receptors on the trypanosome surface use these, or other mechanisms, to ensure that their binding sites are accessible to large macromolecular ligands.

The presence of surface accessible receptors on the bloodstream trypanosomes has consequences, as a receptor that is accessible to a large macromolecular ligand such as HpHb, will also be accessible to an immunoglobin (~150 kDa). In the epimastigote developmental form, this is not a concern as tsetse flies lack an adaptive immune system, allowing HpHbR to be distributed over the entire surface of the cell at high levels [12]. However, in the bloodstream form, exposure of receptors to the serum will put the cells at danger of immune clearance. To avoid this, the cells have evolved significantly lower receptor expression levels [25] together with adopting a predominant localization in the protected environment of the flagellar pocket [1, 29], thereby reducing their likelihood of detection and clearance. In addition, bloodstream form trypanosomes swim forward, creating forces of hydrodynamic flow across their surface, pushing attached ligands towards the flagellar pocket [52]. This has been demonstrated to aid the clearance of antibodies attached to VSG [52, 53], and if receptors are found on the trypanosome surface, their complexes with antibodies will also be swept towards the flagellar pocket for clearance. A similar arrangement, with small quantities of receptors predominantly clustered at the flagellar pocket, is also likely to be seen for other trypanosome bloodstream receptors.

A combination of recent studies of trypanosome HpHbRs have, therefore, shown how one receptor has evolved over eons, adapting to the unique conditions on the cell surface as trypanosome species have diversified. Differences in structure and function between the epimastigote-expressed Hb receptor of T. *congolense* and the bloodstream-form expressed HpHb receptor of *T*. *brucei* have highlighted the different pressures on a receptor that functions within these different surface coats. Future studies will show whether other trypanosome receptors meet these challenges in the same way or accomplish the same goals through different adaptations.
